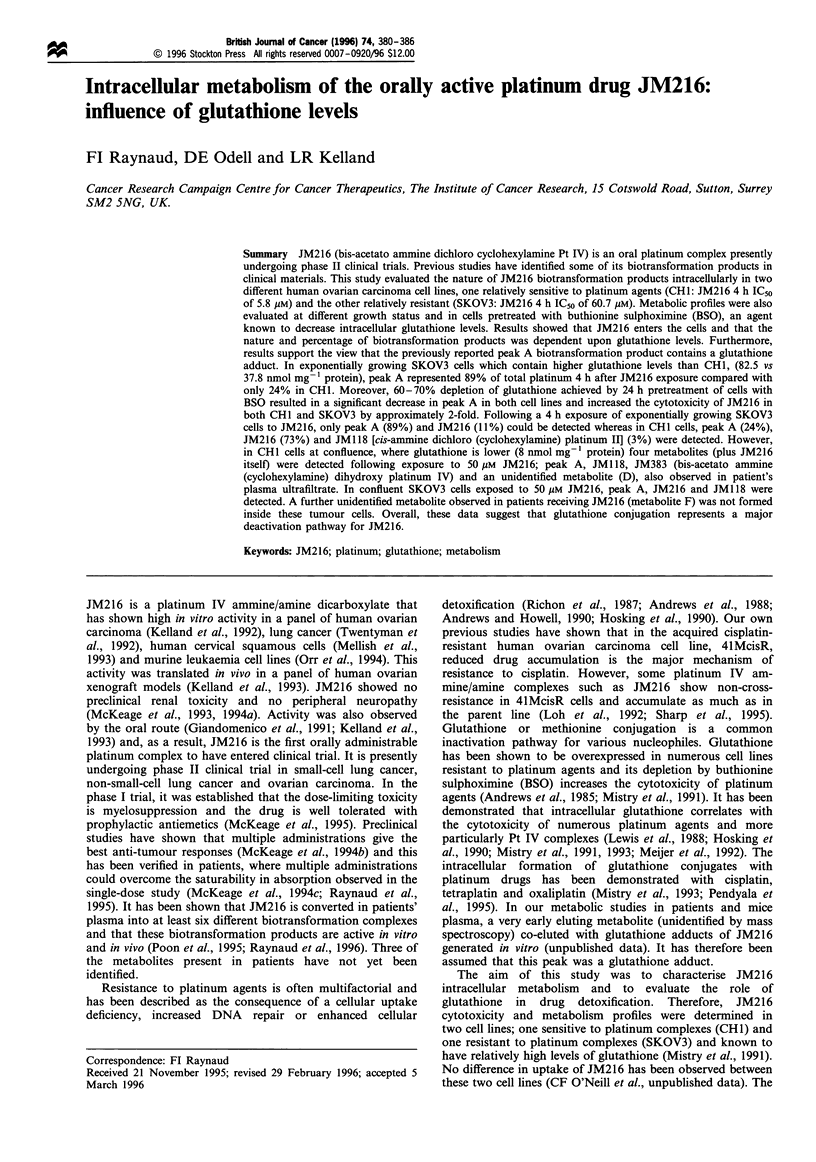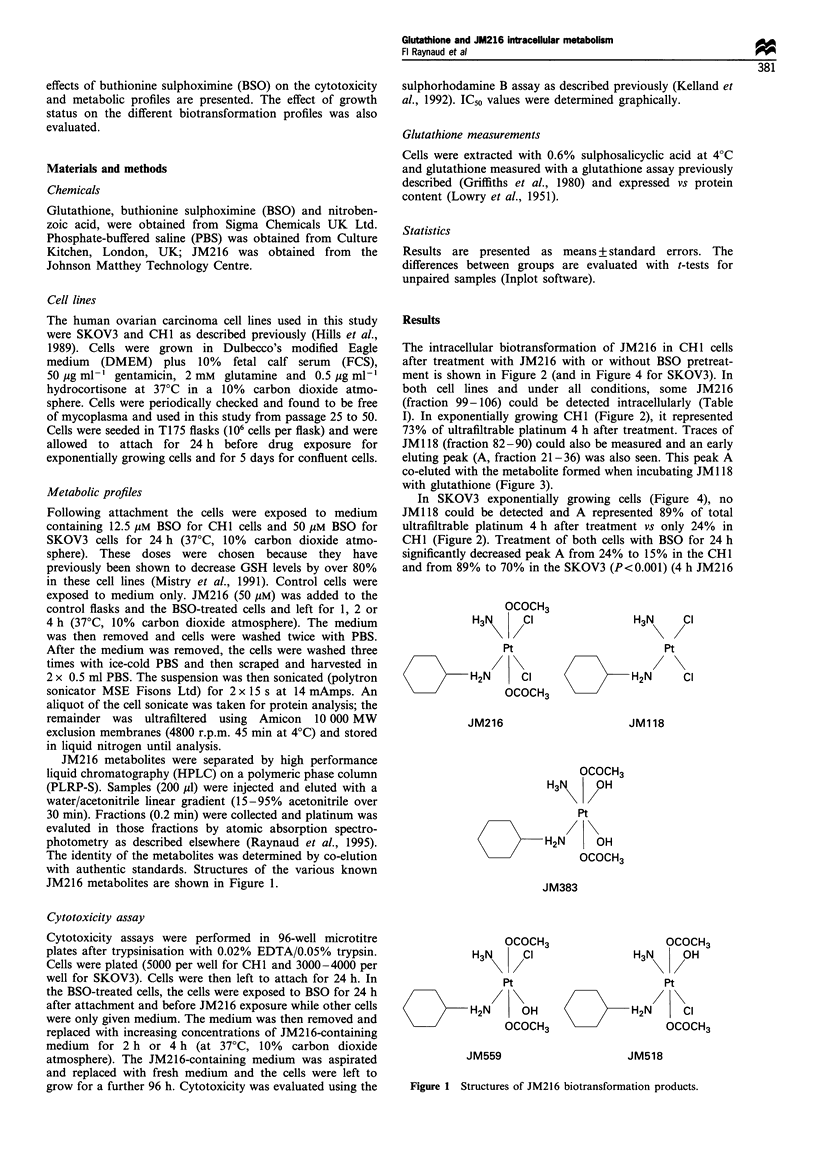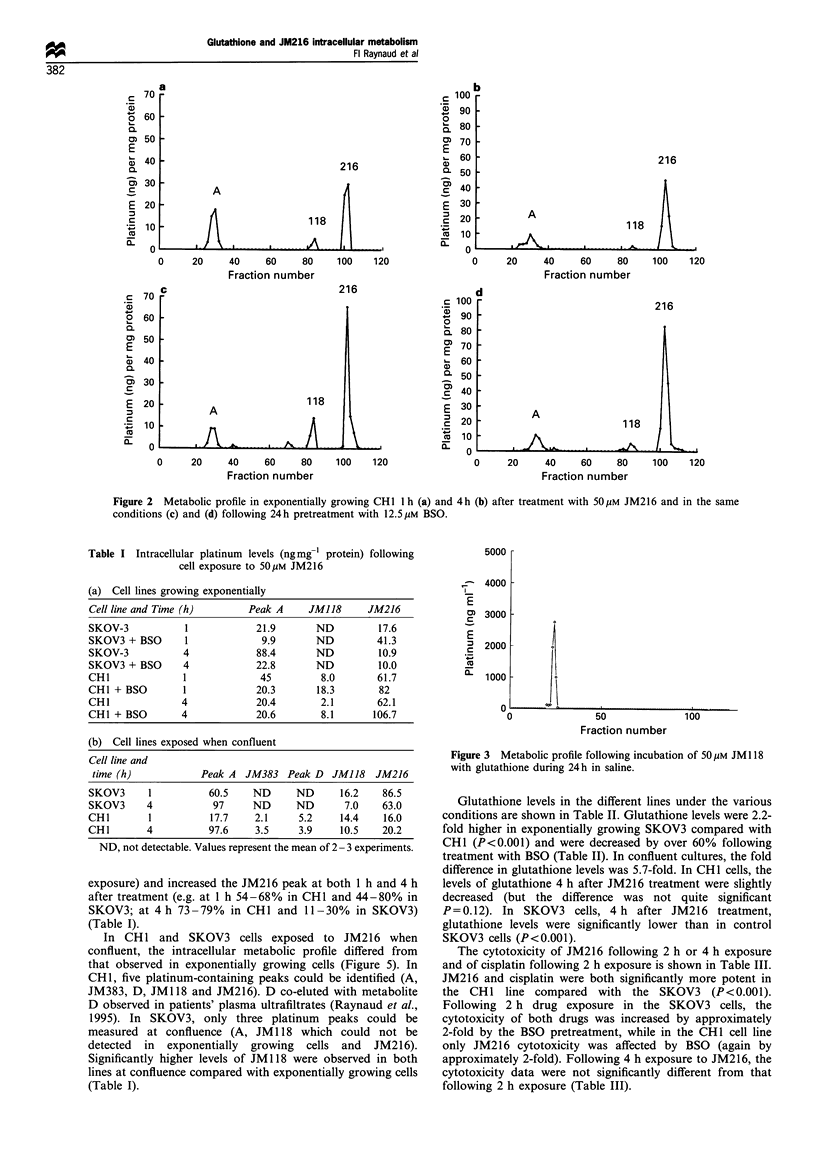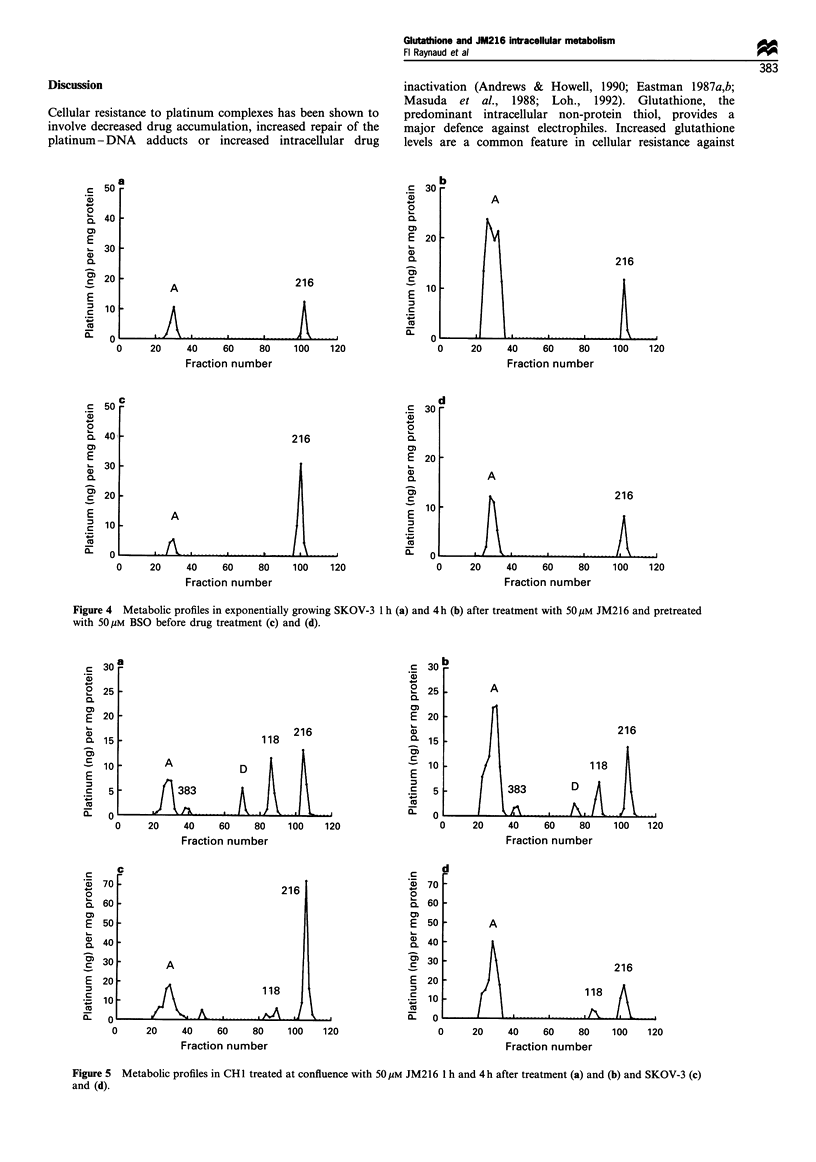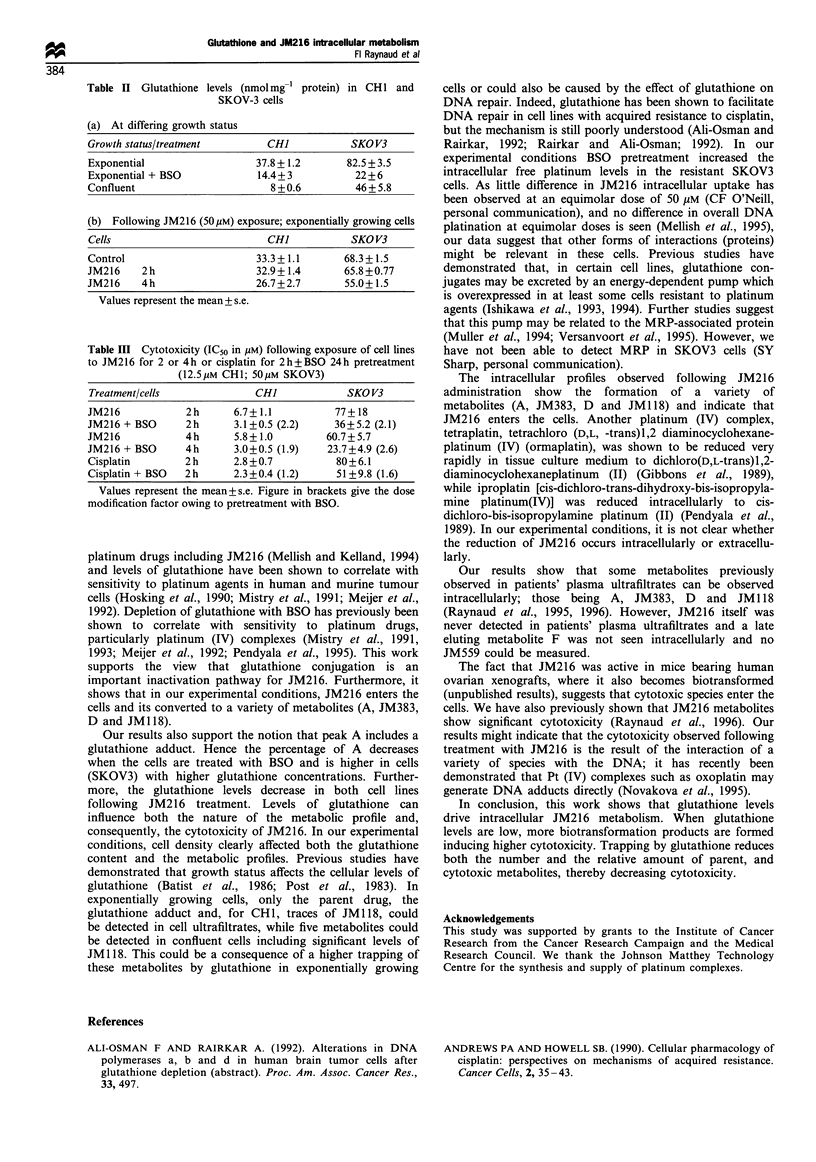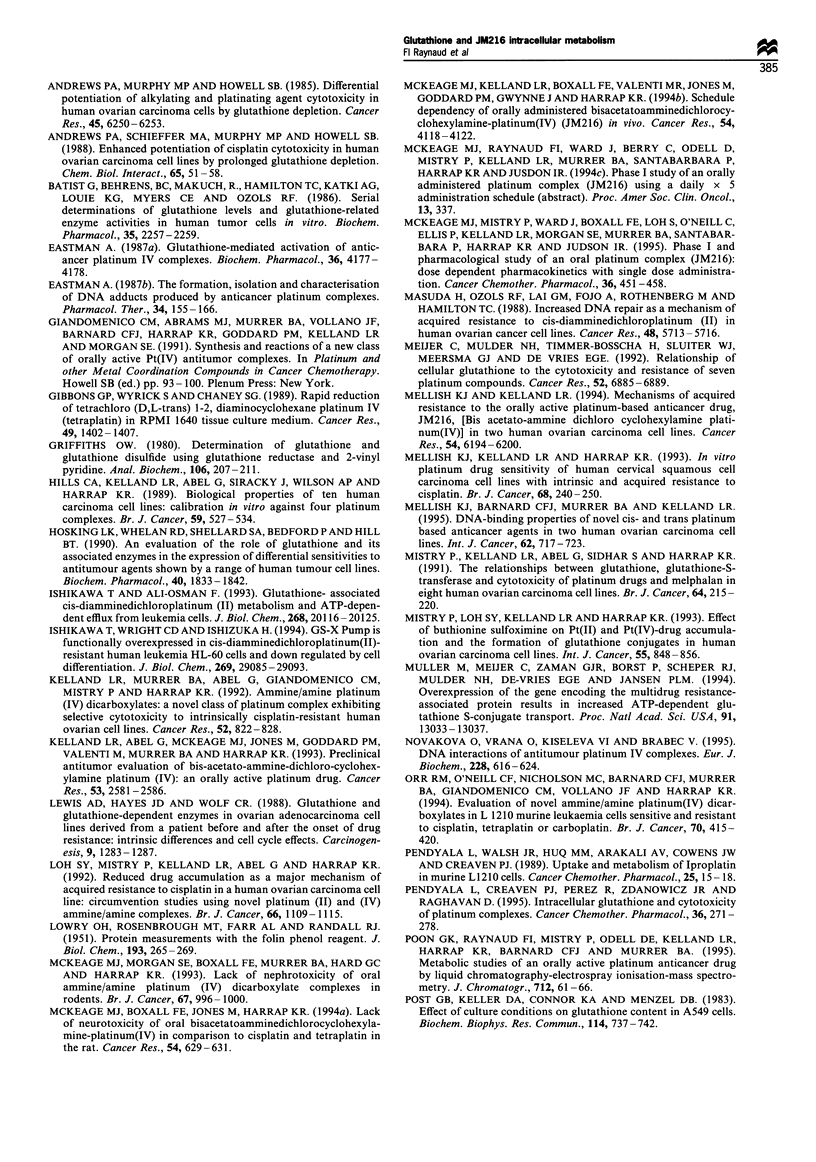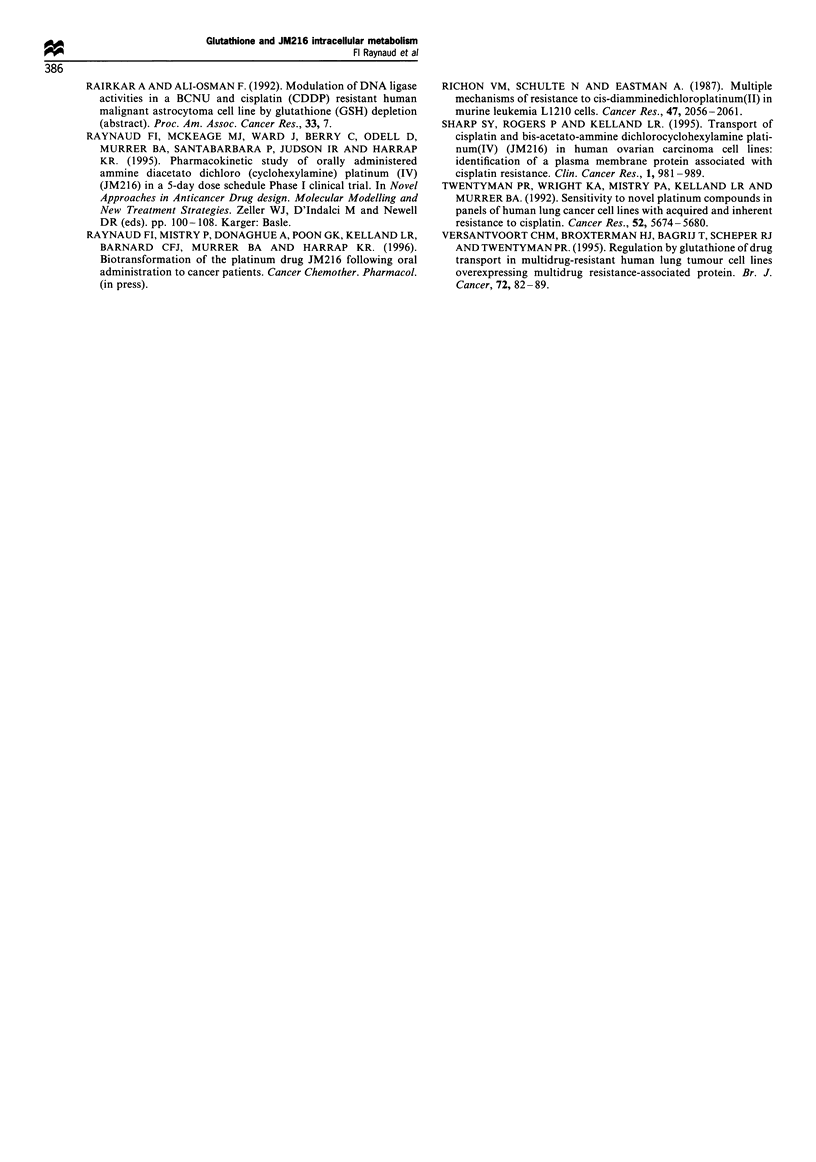# Intracellular metabolism of the orally active platinum drug JM216: influence of glutathione levels.

**DOI:** 10.1038/bjc.1996.369

**Published:** 1996-08

**Authors:** F. I. Raynaud, D. E. Odell, L. R. Kelland

**Affiliations:** Cancer Research Campaign Centre for Cancer Therapeutics, Institute of Cancer Research, Sutton, Surrey, UK.

## Abstract

JM216 (bis-acetato ammine dichloro cyclohexylamine Pt IV) is an oral platinum complex presently undergoing phase II clinical trials. Previous studies have identified some of its biotransformation products in clinical materials. This study evaluated the nature of JM216 biotransformation products intracellularly in two different human ovarian carcinoma cell lines, one relatively sensitive to platinum agents (CH1: JM216 4 h IC50 of 5.8 microM) and the other relatively resistant (SKOV3: JM216 4 h IC50 of 60.7 microM). Metabolic profiles were also evaluated at different growth status and in cells pretreated with buthionine sulphoximine (BSO), an agent known to decrease intracellular glutathione levels. Results showed that JM216 enters the cells and that the nature and percentage of biotransformation products was dependent upon glutathione levels. Furthermore, results support the view that the previously reported peak A biotransformation product contains a glutathione adduct. In exponentially growing SKOV3 cells which contain higher glutathione levels than CH1, (82.5 vs 37.8 nmol mg-1 protein), peak A represented 89% of total platinum 4 h after JM216 exposure compared with only 24% in CH1. Moreover, 60-70% depletion of glutathione achieved by 24 h pretreatment of cells with BSO resulted in a significant decrease in peak A in both cell lines and increased the cytotoxicity of JM216 in both CH1 and SKOV3 by approximately 2-fold. Following a 4 h exposure of exponentially growing SKOV3 cells to JM216, only peak A (89%) and JM216 (11%) could be detected whereas in CH1 cells, peak A (24%), JM216 (73%) and JM118 [cis-ammine dichloro (cyclohexylamine) platinum II] (3%) were detected. However, in CH1 cells at confluence, where glutathione is lower (8 nmol mg-1 protein) four metabolites (plus JM216 itself) were detected following exposure to 50 microM JM216; peak A, JM118, JM383 (bis-acetato ammine (cyclohexylamine) dihydroxy platinum IV) and an unidentified metabolite (D), also observed in patient's plasma ultrafiltrate. In confluent SKOV3 cells exposed to 50 microM JM216, peak A, JM216 and JM118 were detected. A further unidentified metabolite observed in patients receiving JM216 (metabolite F) was not formed inside these tumour cells. Overall, these data suggest that glutathione conjugation represents a major deactivation pathway for JM216.


British Journal of Cancer (1996) 74, 380-386
© 1996 Stockton Press All rights reserved 0007-0920/96 $12.00

### Intracellular metabolism of the orally active platinum drug JM216: influence of glutathione levels

FI Raynaud, DE Odell and LR Kelland

*Cancer Research Campaign Centre for Cancer Therapeutics, The Institute of Cancer Research, 15 Cotswold Road, Sutton, Surrey SM2 5NG, UK.*

**Summary** JM216 (bis-acetato ammine dichloro cyclohexylamine Pt IV) is an oral platinum complex presently undergoing phase II clinical trials. Previous studies have identified some of its biotransformation products in clinical materials. This study evaluated the nature of JM216 biotransformation products intracellularly in two different human ovarian carcinoma cell lines, one relatively sensitive to platinum agents (CH1: JM216 4 h $IC_{50}$ of 5.8 $\mu$M) and the other relatively resistant (SKOV3: JM216 4 h $IC_{50}$ of 60.7 $\mu$M). Metabolic profiles were also evaluated at different growth status and in cells pretreated with buthionine sulphoximine (BSO), an agent known to decrease intracellular glutathione levels. Results showed that JM216 enters the cells and that the nature and percentage of biotransformation products was dependent upon glutathione levels. Furthermore, results support the view that the previously reported peak A biotransformation product contains a glutathione adduct. In exponentially growing SKOV3 cells which contain higher glutathione levels than CH1, (82.5 *vs* 37.8 nmol $mg^{-1}$ protein), peak A represented 89% of total platinum 4 h after JM216 exposure compared with only 24% in CH1. Moreover, 60-70% depletion of glutathione achieved by 24 h pretreatment of cells with BSO resulted in a significant decrease in peak A in both cell lines and increased the cytotoxicity of JM216 in both CH1 and SKOV3 by approximately 2-fold. Following a 4 h exposure of exponentially growing SKOV3 cells to JM216, only peak A (89%) and JM216 (11%) could be detected whereas in CH1 cells, peak A (24%), JM216 (73%) and JM118 [*cis*-ammine dichloro (cyclohexylamine) platinum II] (3%) were detected. However, in CH1 cells at confluence, where glutathione is lower (8 nmol $mg^{-1}$ protein) four metabolites (plus JM216 itself) were detected following exposure to 50 $\mu$M JM216; peak A, JM118, JM383 (bis-acetato ammine (cyclohexylamine) dihydroxy platinum IV) and an unidentified metabolite (D), also observed in patient's plasma ultrafiltrate. In confluent SKOV3 cells exposed to 50 $\mu$M JM216, peak A, JM216 and JM118 were detected. A further unidentified metabolite observed in patients receiving JM216 (metabolite F) was not formed inside these tumour cells. Overall, these data suggest that glutathione conjugation represents a major deactivation pathway for JM216.

**Keywords:** JM216; platinum; glutathione; metabolism

JM216 is a platinum IV ammine/amine dicarboxylate that has shown high *in vitro* activity in a panel of human ovarian carcinoma (Kelland *et al.*, 1992), lung cancer (Twentyman *et al.*, 1992), human cervical squamous cells (Mellish *et al.*, 1993) and murine leukaemia cell lines (Orr *et al.*, 1994). This activity was translated *in vivo* in a panel of human ovarian xenograft models (Kelland *et al.*, 1993). JM216 showed no preclinical renal toxicity and no peripheral neuropathy (McKeage *et al.*, 1993, 1994*a*). Activity was also observed by the oral route (Giandomenico *et al.*, 1991; Kelland *et al.*, 1993) and, as a result, JM216 is the first orally administrable platinum complex to have entered clinical trial. It is presently undergoing phase II clinical trial in small-cell lung cancer, non-small-cell lung cancer and ovarian carcinoma. In the phase I trial, it was established that the dose-limiting toxicity is myelosuppression and the drug is well tolerated with prophylactic antiemetics (McKeage *et al.*, 1995). Preclinical studies have shown that multiple administrations give the best anti-tumour responses (McKeage *et al.*, 1994*b*) and this has been verified in patients, where multiple administrations could overcome the saturability in absorption observed in the single-dose study (McKeage *et al.*, 1994*c*; Raynaud *et al.*, 1995). It has been shown that JM216 is converted in patients' plasma into at least six different biotransformation complexes and that these biotransformation products are active *in vitro* and *in vivo* (Poon *et al.*, 1995; Raynaud *et al.*, 1996). Three of the metabolites present in patients have not yet been identified.

Resistance to platinum agents is often multifactorial and has been described as the consequence of a cellular uptake deficiency, increased DNA repair or enhanced cellular detoxification (Richon *et al.*, 1987; Andrews *et al.*, 1988; Andrews and Howell, 1990; Hosking *et al.*, 1990). Our own previous studies have shown that in the acquired cisplatin-resistant human ovarian carcinoma cell line, 41McisR, reduced drug accumulation is the major mechanism of resistance to cisplatin. However, some platinum IV ammine/amine complexes such as JM216 show non-cross-resistance in 41McisR cells and accumulate as much as in the parent line (Loh *et al.*, 1992; Sharp *et al.*, 1995). Glutathione or methionine conjugation is a common inactivation pathway for various nucleophiles. Glutathione has been shown to be overexpressed in numerous cell lines resistant to platinum agents and its depletion by buthionine sulphoximine (BSO) increases the cytotoxicity of platinum agents (Andrews *et al.*, 1985; Mistry *et al.*, 1991). It has been demonstrated that intracellular glutathione correlates with the cytotoxicity of numerous platinum agents and more particularly Pt IV complexes (Lewis *et al.*, 1988; Hosking *et al.*, 1990; Mistry *et al.*, 1991, 1993; Meijer *et al.*, 1992). The intracellular formation of glutathione conjugates with platinum drugs has been demonstrated with cisplatin, tetraplatin and oxaliplatin (Mistry *et al.*, 1993; Pendyala *et al.*, 1995). In our metabolic studies in patients and mice plasma, a very early eluting metabolite (unidentified by mass spectroscopy) co-eluted with glutathione adducts of JM216 generated *in vitro* (unpublished data). It has therefore been assumed that this peak was a glutathione adduct.

The aim of this study was to characterise JM216 intracellular metabolism and to evaluate the role of glutathione in drug detoxification. Therefore, JM216 cytotoxicity and metabolism profiles were determined in two cell lines; one sensitive to platinum complexes (CH1) and one resistant to platinum complexes (SKOV3) and known to have relatively high levels of glutathione (Mistry *et al.*, 1991). No difference in uptake of JM216 has been observed between these two cell lines (CF O'Neill *et al.*, unpublished data). The

Correspondence: FI Raynaud
Received 21 November 1995; revised 29 February 1996; accepted 5 March 1996

effects of buthionine sulphoximine (BSO) on the cytotoxicity and metabolic profiles are presented. The effect of growth status on the different biotransformation profiles was also evaluated.

### Materials and methods

### *Chemicals*

Glutathione, buthionine sulphoximine (BSO) and nitrobenzoic acid, were obtained from Sigma Chemicals UK Ltd. Phosphate-buffered saline (PBS) was obtained from Culture Kitchen, London, UK; JM216 was obtained from the Johnson Matthey Technology Centre.

### *Cell lines*

The human ovarian carcinoma cell lines used in this study were SKOV3 and CH1 as described previously (Hills *et al.*, 1989). Cells were grown in Dulbecco's modified Eagle medium (DMEM) plus 10% fetal calf serum (FCS), 50 $\mu$g ml$^{-1}$ gentamicin, 2 mM glutamine and 0.5 $\mu$g ml$^{-1}$ hydrocortisone at 37°C in a 10% carbon dioxide atmosphere. Cells were periodically checked and found to be free of mycoplasma and used in this study from passage 25 to 50. Cells were seeded in T175 flasks ($10^6$ cells per flask) and were allowed to attach for 24 h before drug exposure for exponentially growing cells and for 5 days for confluent cells.

### *Metabolic profiles*

Following attachment the cells were exposed to medium containing 12.5 $\mu$M BSO for CH1 cells and 50 $\mu$M BSO for SKOV3 cells for 24 h (37°C, 10% carbon dioxide atmosphere). These doses were chosen because they have previously been shown to decrease GSH levels by over 80% in these cell lines (Mistry *et al.*, 1991). Control cells were exposed to medium only. JM216 (50 $\mu$M) was added to the control flasks and the BSO-treated cells and left for 1, 2 or 4 h (37°C, 10% carbon dioxide atmosphere). The medium was then removed and cells were washed twice with PBS. After the medium was removed, the cells were washed three times with ice-cold PBS and then scraped and harvested in 2 × 0.5 ml PBS. The suspension was then sonicated (polytron sonicator MSE Fisons Ltd) for 2 × 15 s at 14 mAmps. An aliquot of the cell sonicate was taken for protein analysis; the remainder was ultrafiltered using Amicon 10 000 MW exclusion membranes (4800 r.p.m. 45 min at 4°C) and stored in liquid nitrogen until analysis.

JM216 metabolites were separated by high performance liquid chromatography (HPLC) on a polymeric phase column (PLRP-S). Samples (200 $\mu$l) were injected and eluted with a water/acetonitrile linear gradient (15–95% acetonitrile over 30 min). Fractions (0.2 min) were collected and platinum was evaluted in those fractions by atomic absorption spectrophotometry as described elsewhere (Raynaud *et al.*, 1995). The identity of the metabolites was determined by co-elution with authentic standards. Structures of the various known JM216 metabolites are shown in Figure 1.

### *Cytotoxicity assay*

Cytotoxicity assays were performed in 96-well microtitre plates after trypsinisation with 0.02% EDTA/0.05% trypsin. Cells were plated (5000 per well for CH1 and 3000–4000 per well for SKOV3). Cells were then left to attach for 24 h. In the BSO-treated cells, the cells were exposed to BSO for 24 h after attachment and before JM216 exposure while other cells were only given medium. The medium was then removed and replaced with increasing concentrations of JM216-containing medium for 2 h or 4 h (at 37°C, 10% carbon dioxide atmosphere). The JM216-containing medium was aspirated and replaced with fresh medium and the cells were left to grow for a further 96 h. Cytotoxicity was evaluated using the sulphorhodamine B assay as described previously (Kelland *et al.*, 1992). $IC_{50}$ values were determined graphically.

### *Glutathione measurements*

Cells were extracted with 0.6% sulphosalicyclic acid at 4°C and glutathione measured with a glutathione assay previously described (Griffiths *et al.*, 1980) and expressed *vs* protein content (Lowry *et al.*, 1951).

### *Statistics*

Results are presented as means ± standard errors. The differences between groups are evaluated with *t*-tests for unpaired samples (Inplot software).

### Results

The intracellular biotransformation of JM216 in CH1 cells after treatment with JM216 with or without BSO pretreatment is shown in Figure 2 (and in Figure 4 for SKOV3). In both cell lines and under all conditions, some JM216 (fraction 99–106) could be detected intracellularly (Table I). In exponentially growing CH1 (Figure 2), it represented 73% of ultrafiltrable platinum 4 h after treatment. Traces of JM118 (fraction 82–90) could also be measured and an early eluting peak (A, fraction 21–36) was also seen. This peak A co-eluted with the metabolite formed when incubating JM118 with glutathione (Figure 3).

In SKOV3 exponentially growing cells (Figure 4), no JM118 could be detected and A represented 89% of total ultrafiltrable platinum 4 h after treatment *vs* only 24% in CH1 (Figure 2). Treatment of both cells with BSO for 24 h significantly decreased peak A from 24% to 15% in the CH1 and from 89% to 70% in the SKOV3 ($P<0.001$) (4 h JM216

JM216

JM118

JM383

JM559

JM518

**Figure 1** Structures of JM216 biotransformation products.

**Figure 2** Metabolic profile in exponentially growing CHI 1 h (**a**) and 4 h (**b**) after treatment with 50 μM JM216 and in the same conditions (**c**) and (**d**) following 24 h pretreatment with 12.5 μM BSO.

**Table I** Intracellular platinum levels (ng $mg^{-1}$ protein) following cell exposure to 50 μM JM216

(a) Cell lines growing exponentially

| *Cell line and Time (h)* | | *Peak A* | *JM118* | *JM216* |
|---|---|---|---|---|
| SKOV-3 | 1 | 21.9 | ND | 17.6 |
| SKOV3 + BSO | 1 | 9.9 | ND | 41.3 |
| SKOV-3 | 4 | 88.4 | ND | 10.9 |
| SKOV3 + BSO | 4 | 22.8 | ND | 10.0 |
| CH1 | 1 | 45 | 8.0 | 61.7 |
| CH1 + BSO | 1 | 20.3 | 18.3 | 82 |
| CH1 | 4 | 20.4 | 2.1 | 62.1 |
| CH1 + BSO | 4 | 20.6 | 8.1 | 106.7 |

(b) Cell lines exposed when confluent

| *Cell line and time (h)* | | *Peak A* | *JM383* | *Peak D* | *JM118* | *JM216* |
|---|---|---|---|---|---|---|
| SKOV3 | 1 | 60.5 | ND | ND | 16.2 | 86.5 |
| SKOV3 | 4 | 97 | ND | ND | 7.0 | 63.0 |
| CH1 | 1 | 17.7 | 2.1 | 5.2 | 14.4 | 16.0 |
| CH1 | 4 | 97.6 | 3.5 | 3.9 | 10.5 | 20.2 |

ND, not detectable. Values represent the mean of 2–3 experiments.

exposure) and increased the JM216 peak at both 1 h and 4 h after treatment (e.g. at 1 h 54–68% in CH1 and 44–80% in SKOV3; at 4 h 73–79% in CH1 and 11–30% in SKOV3) (Table I).

In CH1 and SKOV3 cells exposed to JM216 when confluent, the intracellular metabolic profile differed from that observed in exponentially growing cells (Figure 5). In CH1, five platinum-containing peaks could be identified (A, JM383, D, JM118 and JM216). D co-eluted with metabolite D observed in patients' plasma ultrafiltrates (Raynaud *et al.*, 1995). In SKOV3, only three platinum peaks could be measured at confluence (A, JM118 which could not be detected in exponentially growing cells and JM216). Significantly higher levels of JM118 were observed in both lines at confluence compared with exponentially growing cells (Table I).

**Figure 3** Metabolic profile following incubation of 50 μM JM118 with glutathione during 24 h in saline.

Glutathione levels in the different lines under the various conditions are shown in Table II. Glutathione levels were 2.2-fold higher in exponentially growing SKOV3 compared with CH1 ($P<0.001$) and were decreased by over 60% following treatment with BSO (Table II). In confluent cultures, the fold difference in glutathione levels was 5.7-fold. In CH1 cells, the levels of glutathione 4 h after JM216 treatment were slightly decreased (but the difference was not quite significant $P=0.12$). In SKOV3 cells, 4 h after JM216 treatment, glutathione levels were significantly lower than in control SKOV3 cells ($P<0.001$).

The cytotoxicity of JM216 following 2 h or 4 h exposure and of cisplatin following 2 h exposure is shown in Table III. JM216 and cisplatin were both significantly more potent in the CH1 line compared with the SKOV3 ($P<0.001$). Following 2 h drug exposure in the SKOV3 cells, the cytotoxicity of both drugs was increased by approximately 2-fold by the BSO pretreatment, while in the CH1 cell line only JM216 cytotoxicity was affected by BSO (again by approximately 2-fold). Following 4 h exposure to JM216, the cytotoxicity data were not significantly different from that following 2 h exposure (Table III).

### Discussion

Cellular resistance to platinum complexes has been shown to involve decreased drug accumulation, increased repair of the platinum–DNA adducts or increased intracellular drug inactivation (Andrews & Howell, 1990; Eastman 1987*a*,*b*; Masuda *et al*., 1988; Loh., 1992). Glutathione, the predominant intracellular non-protein thiol, provides a major defence against electrophiles. Increased glutathione levels are a common feature in cellular resistance against

**Figure 4** Metabolic profiles in exponentially growing SKOV-3 1 h (**a**) and 4 h (**b**) after treatment with 50 $\mu$M JM216 and pretreated with 50 $\mu$M BSO before drug treatment (**c**) and (**d**).

**Figure 5** Metabolic profiles in CH1 treated at confluence with 50 $\mu$M JM216 1 h and 4 h after treatment (**a**) and (**b**) and SKOV-3 (**c**) and (**d**).

**Table II** Glutathione levels (nmol $mg^{-1}$ protein) in CH1 and SKOV-3 cells

(a) At differing growth status

| *Growth status/treatment* | *CH1* | *SKOV3* |
|---|---|---|
| Exponential | 37.8±1.2 | 82.5±3.5 |
| Exponential + BSO | 14.4±3 | 22±6 |
| Confluent | 8±0.6 | 46±5.8 |

(b) Following JM216 (50 μM) exposure; exponentially growing cells

| *Cells* | | *CH1* | *SKOV3* |
|---|---|---|---|
| Control | | 33.3±1.1 | 68.3±1.5 |
| JM216 | 2h | 32.9±1.4 | 65.8±0.77 |
| JM216 | 4h | 26.7±2.7 | 55.0±1.5 |

Values represent the mean±s.e.

**Table III** Cytotoxicity ($IC_{50}$ in μM) following exposure of cell lines to JM216 for 2 or 4h or cisplatin for 2h±BSO 24h pretreatment (12.5 μM CH1; 50 μM SKOV3)

| *Treatment/cells* | | *CH1* | *SKOV3* |
|---|---|---|---|
| JM216 | 2h | 6.7±1.1 | 77±18 |
| JM216 + BSO | 2h | 3.1±0.5 (2.2) | 36±5.2 (2.1) |
| JM216 | 4h | 5.8±1.0 | 60.7±5.7 |
| JM216 + BSO | 4h | 3.0±0.5 (1.9) | 23.7±4.9 (2.6) |
| Cisplatin | 2h | 2.8±0.7 | 80±6.1 |
| Cisplatin + BSO | 2h | 2.3±0.4 (1.2) | 51±9.8 (1.6) |

Values represent the mean±s.e. Figure in brackets give the dose modification factor owing to pretreatment with BSO.

platinum drugs including JM216 (Mellish and Kelland, 1994) and levels of glutathione have been shown to correlate with sensitivity to platinum agents in human and murine tumour cells (Hosking *et al.*, 1990; Mistry *et al.*, 1991; Meijer *et al.*, 1992). Depletion of glutathione with BSO has previously been shown to correlate with sensitivity to platinum drugs, particularly platinum (IV) complexes (Mistry *et al.*, 1991, 1993; Meijer *et al.*, 1992; Pendyala *et al.*, 1995). This work supports the view that glutathione conjugation is an important inactivation pathway for JM216. Furthermore, it shows that in our experimental conditions, JM216 enters the cells and its converted to a variety of metabolites (A, JM383, D and JM118).

Our results also support the notion that peak A includes a glutathione adduct. Hence the percentage of A decreases when the cells are treated with BSO and is higher in cells (SKOV3) with higher glutathione concentrations. Furthermore, the glutathione levels decrease in both cell lines following JM216 treatment. Levels of glutathione can influence both the nature of the metabolic profile and, consequently, the cytotoxicity of JM216. In our experimental conditions, cell density clearly affected both the glutathione content and the metabolic profiles. Previous studies have demonstrated that growth status affects the cellular levels of glutathione (Batist *et al.*, 1986; Post *et al.*, 1983). In exponentially growing cells, only the parent drug, the glutathione adduct and, for CH1, traces of JM118, could be detected in cell ultrafiltrates, while five metabolites could be detected in confluent cells including significant levels of JM118. This could be a consequence of a higher trapping of these metabolites by glutathione in exponentially growing cells or could also be caused by the effect of glutathione on DNA repair. Indeed, glutathione has been shown to facilitate DNA repair in cell lines with acquired resistance to cisplatin, but the mechanism is still poorly understood (Ali-Osman and Rairkar, 1992; Rairkar and Ali-Osman; 1992). In our experimental conditions BSO pretreatment increased the intracellular free platinum levels in the resistant SKOV3 cells. As little difference in JM216 intracellular uptake has been observed at an equimolar dose of 50 μM (CF O'Neill, personal communication), and no difference in overall DNA platination at equimolar doses is seen (Mellish *et al.*, 1995), our data suggest that other forms of interactions (proteins) might be relevant in these cells. Previous studies have demonstrated that, in certain cell lines, glutathione conjugates may be excreted by an energy-dependent pump which is overexpressed in at least some cells resistant to platinum agents (Ishikawa *et al.*, 1993, 1994). Further studies suggest that this pump may be related to the MRP-associated protein (Muller *et al.*, 1994; Versanvoort *et al.*, 1995). However, we have not been able to detect MRP in SKOV3 cells (SY Sharp, personal communication).

The intracellular profiles observed following JM216 administration show the formation of a variety of metabolites (A, JM383, D and JM118) and indicate that JM216 enters the cells. Another platinum (IV) complex, tetraplatin, tetrachloro (D,L, -trans)1,2 diaminocyclohexane-platinum (IV) (ormaplatin), was shown to be reduced very rapidly in tissue culture medium to dichloro(D,L-trans)1,2-diaminocyclohexaneplatinum (II) (Gibbons *et al.*, 1989), while iproplatin [cis-dichloro-trans-dihydroxy-bis-isopropylamine platinum(IV)] was reduced intracellularly to cis-dichloro-bis-isopropylamine platinum (II) (Pendyala *et al.*, 1989). In our experimental conditions, it is not clear whether the reduction of JM216 occurs intracellularly or extracellularly.

Our results show that some metabolites previously observed in patients' plasma ultrafiltrates can be observed intracellularly; those being A, JM383, D and JM118 (Raynaud *et al.*, 1995, 1996). However, JM216 itself was never detected in patients' plasma ultrafiltrates and a late eluting metabolite F was not seen intracellularly and no JM559 could be measured.

The fact that JM216 was active in mice bearing human ovarian xenografts, where it also becomes biotransformed (unpublished results), suggests that cytotoxic species enter the cells. We have also previously shown that JM216 metabolites show significant cytotoxicity (Raynaud *et al.*, 1996). Our results might indicate that the cytotoxicity observed following treatment with JM216 is the result of the interaction of a variety of species with the DNA; it has recently been demonstrated that Pt (IV) complexes such as oxoplatin may generate DNA adducts directly (Novakova *et al.*, 1995).

In conclusion, this work shows that glutathione levels drive intracellular JM216 metabolism. When glutathione levels are low, more biotransformation products are formed inducing higher cytotoxicity. Trapping by glutathione reduces both the number and the relative amount of parent, and cytotoxic metabolites, thereby decreasing cytotoxicity.

**Acknowledgements**
This study was supported by grants to the Institute of Cancer Research from the Cancer Research Campaign and the Medical Research Council. We thank the Johnson Matthey Technology Centre for the synthesis and supply of platinum complexes.

### References

ALI-OSMAN F AND RAIRKAR A. (1992). Alterations in DNA polymerases a, b and d in human brain tumor cells after glutathione depletion (abstract). *Proc. Am. Assoc. Cancer Res.*, **33**, 497.

ANDREWS PA AND HOWELL SB. (1990). Cellular pharmacology of cisplatin: perspectives on mechanisms of acquired resistance. *Cancer Cells*, **2**, 35–43.

ANDREWS PA, MURPHY MP AND HOWELL SB. (1985). Differential potentiation of alkylating and platinating agent cytotoxicity in human ovarian carcinoma cells by glutathione depletion. *Cancer Res.*, **45**, 6250–6253.

ANDREWS PA, SCHIEFFER MA, MURPHY MP AND HOWELL SB. (1988). Enhanced potentiation of cisplatin cytotoxicity in human ovarian carcinoma cell lines by prolonged glutathione depletion. *Chem. Biol. Interact.*, **65**, 51–58.

BATIST G, BEHRENS, BC, MAKUCH, R., HAMILTON TC, KATKI AG, LOUIE KG, MYERS CE AND OZOLS RF. (1986). Serial determinations of glutathione levels and glutathione-related enzyme activities in human tumor cells *in vitro*. *Biochem. Pharmacol.*, **35**, 2257–2259.

EASTMAN A. (1987*a*). Glutathione-mediated activation of anticancer platinum IV complexes. *Biochem. Pharmacol.*, **36**, 4177–4178.

EASTMAN A. (1987*b*). The formation, isolation and characterisation of DNA adducts produced by anticancer platinum complexes. *Pharmacol. Ther.*, **34**, 155–166.

GIANDOMENICO CM, ABRAMS MJ, MURRER BA, VOLLANO JF, BARNARD CFJ, HARRAP KR, GODDARD PM, KELLAND LR AND MORGAN SE. (1991). Synthesis and reactions of a new class of orally active Pt(IV) antitumor complexes. In *Platinum and other Metal Coordination Compounds in Cancer Chemotherapy.* Howell SB (ed.) pp. 93–100. Plenum Press: New York.

GIBBONS GP, WYRICK S AND CHANEY SG. (1989). Rapid reduction of tetrachloro (D,L-trans) 1-2, diaminocyclohexane platinum IV (tetraplatin) in RPMI 1640 tissue culture medium. *Cancer Res.*, **49**, 1402–1407.

GRIFFITHS OW. (1980). Determination of glutathione and glutathione disulfide using glutathione reductase and 2-vinyl pyridine. *Anal. Biochem.*, **106**, 207–211.

HILLS CA, KELLAND LR, ABEL G, SIRACKY J, WILSON AP AND HARRAP KR. (1989). Biological properties of ten human carcinoma cell lines: calibration *in vitro* against four platinum complexes. *Br. J. Cancer*, **59**, 527–534.

HOSKING LK, WHELAN RD, SHELLARD SA, BEDFORD P AND HILL BT. (1990). An evaluation of the role of glutathione and its associated enzymes in the expression of differential sensitivities to antitumour agents shown by a range of human tumour cell lines. *Biochem. Pharmacol.*, **40**, 1833–1842.

ISHIKAWA T AND ALI-OSMAN F. (1993). Glutathione- associated cis-diamminedichloroplatinum (II) metabolism and ATP-dependent efflux from leukemia cells. *J. Biol. Chem.*, **268**, 20116–20125.

ISHIKAWA T, WRIGHT CD AND ISHIZUKA H. (1994). GS-X Pump is functionally overexpressed in cis-diamminedichloroplatinum(II)-resistant human leukemia HL-60 cells and down regulated by cell differentiation. *J. Biol. Chem.*, **269**, 29085–29093.

KELLAND LR, MURRER BA, ABEL G, GIANDOMENICO CM, MISTRY P AND HARRAP KR. (1992). Ammine/amine platinum (IV) dicarboxylates: a novel class of platinum complex exhibiting selective cytotoxicity to intrinsically cisplatin-resistant human ovarian cell lines. *Cancer Res.*, **52**, 822–828.

KELLAND LR, ABEL G, MCKEAGE MJ, JONES M, GODDARD PM, VALENTI M, MURRER BA AND HARRAP KR. (1993). Preclinical antitumor evaluation of bis-acetato-ammine-dichloro-cyclohexylamine platinum (IV): an orally active platinum drug. *Cancer Res.*, **53**, 2581–2586.

LEWIS AD, HAYES JD AND WOLF CR. (1988). Glutathione and glutathione-dependent enzymes in ovarian adenocarcinoma cell lines derived from a patient before and after the onset of drug resistance: intrinsic differences and cell cycle effects. *Carcinogenesis*, **9**, 1283–1287.

LOH SY, MISTRY P, KELLAND LR, ABEL G AND HARRAP KR. (1992). Reduced drug accumulation as a major mechanism of acquired resistance to cisplatin in a human ovarian carcinoma cell line: circumvention studies using novel platinum (II) and (IV) ammine/amine complexes. *Br. J. Cancer*, **66**, 1109–1115.

LOWRY OH, ROSENBROUGH MT, FARR AL AND RANDALL RJ. (1951). Protein measurements with the folin phenol reagent. *J. Biol. Chem.*, **193**, 265–269.

MCKEAGE MJ, MORGAN SE, BOXALL FE, MURRER BA, HARD GC AND HARRAP KR. (1993). Lack of nephrotoxicity of oral ammine/amine platinum (IV) dicarboxylate complexes in rodents. *Br. J. Cancer*, **67**, 996–1000.

MCKEAGE MJ, BOXALL FE, JONES M, HARRAP KR. (1994*a*). Lack of neurotoxicity of oral bisacetatoamminedichlorocyclohexylamine-platinum(IV) in comparison to cisplatin and tetraplatin in the rat. *Cancer Res.*, **54**, 629–631.

MCKEAGE MJ, KELLAND LR, BOXALL FE, VALENTI MR, JONES M, GODDARD PM, GWYNNE J AND HARRAP KR. (1994*b*). Schedule dependency of orally administered bisacetatoamminedichlorocyclohexylamine-platinum(IV) (JM216) *in vivo*. *Cancer Res.*, **54**, 4118–4122.

MCKEAGE MJ, RAYNAUD FI, WARD J, BERRY C, ODELL D, MISTRY P, KELLAND LR, MURRER BA, SANTABARBARA P, HARRAP KR AND JUSDON IR. (1994*c*). Phase I study of an orally administered platinum complex (JM216) using a daily × 5 administration schedule (abstract). *Proc. Amer Soc. Clin. Oncol.*, **13**, 337.

MCKEAGE MJ, MISTRY P, WARD J, BOXALL FE, LOH S, O'NEILL C, ELLIS P, KELLAND LR, MORGAN SE, MURRER BA, SANTABARBARA P, HARRAP KR AND JUDSON IR. (1995). Phase I and pharmacological study of an oral platinum complex (JM216): dose dependent pharmacokinetics with single dose administration. *Cancer Chemother. Pharmacol.*, **36**, 451–458.

MASUDA H, OZOLS RF, LAI GM, FOJO A, ROTHENBERG M AND HAMILTON TC. (1988). Increased DNA repair as a mechanism of acquired resistance to cis-diamminedichloroplatinum (II) in human ovarian cancer cell lines. *Cancer Res.*, **48**, 5713–5716.

MEIJER C, MULDER NH, TIMMER-BOSSCHA H, SLUITER WJ, MEERSMA GJ AND DE VRIES EGE. (1992). Relationship of cellular glutathione to the cytotoxicity and resistance of seven platinum compounds. *Cancer Res.*, **52**, 6885–6889.

MELLISH KJ AND KELLAND LR. (1994). Mechanisms of acquired resistance to the orally active platinum-based anticancer drug, JM216, [Bis acetato-ammine dichloro cyclohexylamine platinum(IV)] in two human ovarian carcinoma cell lines. *Cancer Res.*, **54**, 6194–6200.

MELLISH KJ, KELLAND LR AND HARRAP KR. (1993). *In vitro* platinum drug sensitivity of human cervical squamous cell carcinoma cell lines with intrinsic and acquired resistance to cisplatin. *Br. J. Cancer*, **68**, 240–250.

MELLISH KJ, BARNARD CFJ, MURRER BA AND KELLAND LR. (1995). DNA-binding properties of novel cis- and trans platinum based anticancer agents in two human ovarian carcinoma cell lines. *Int. J. Cancer*, **62**, 717–723.

MISTRY P., KELLAND LR, ABEL G, SIDHAR S AND HARRAP KR. (1991). The relationships between glutathione, glutathione-S-transferase and cytotoxicity of platinum drugs and melphalan in eight human ovarian carcinoma cell lines. *Br. J. Cancer*, **64**, 215–220.

MISTRY P, LOH SY, KELLAND LR AND HARRAP KR. (1993). Effect of buthionine sulfoximine on Pt(II) and Pt(IV)-drug accumulation and the formation of glutathione conjugates in human ovarian carcinoma cell lines. *Int. J. Cancer*, **55**, 848–856.

MULLER M, MEIJER C, ZAMAN GJR, BORST P, SCHEPER RJ, MULDER NH, DE-VRIES EGE AND JANSEN PLM. (1994). Overexpression of the gene encoding the multidrug resistance-associated protein results in increased ATP-dependent glutathione S-conjugate transport. *Proc. Natl Acad. Sci. USA*, **91**, 13033–13037.

NOVAKOVA O, VRANA O, KISELEVA VI AND BRABEC V. (1995). DNA interactions of antitumour platinum IV complexes. *Eur. J. Biochem.*, **228**, 616–624.

ORR RM, O'NEILL CF, NICHOLSON MC, BARNARD CFJ, MURRER BA, GIANDOMENICO CM, VOLLANO JF AND HARRAP KR. (1994). Evaluation of novel ammine/amine platinum(IV) dicarboxylates in L 1210 murine leukaemia cells sensitive and resistant to cisplatin, tetraplatin or carboplatin. *Br. J. Cancer*, **70**, 415–420.

PENDYALA L, WALSH JR, HUQ MM, ARAKALI AV, COWENS JW AND CREAVEN PJ. (1989). Uptake and metabolism of Iproplatin in murine L1210 cells. *Cancer Chemother. Pharmacol.*, **25**, 15–18.

PENDYALA L, CREAVEN PJ, PEREZ R, ZDANOWICZ JR AND RAGHAVAN D. (1995). Intracellular glutathione and cytotoxicity of platinum complexes. *Cancer Chemother. Pharmacol.*, **36**, 271–278.

POON GK, RAYNAUD FI, MISTRY P, ODELL DE, KELLAND LR, HARRAP KR, BARNARD CFJ AND MURRER BA. (1995). Metabolic studies of an orally active platinum anticancer drug by liquid chromatography-electrospray ionisation-mass spectrometry. *J. Chromatogr.*, **712**, 61–66.

POST GB, KELLER DA, CONNOR KA AND MENZEL DB. (1983). Effect of culture conditions on glutathione content in A549 cells. *Biochem. Biophys. Res. Commun.*, **114**, 737–742.

RAIRKAR A AND ALI-OSMAN F. (1992). Modulation of DNA ligase activities in a BCNU and cisplatin (CDDP) resistant human malignant astrocytoma cell line by glutathione (GSH) depletion (abstract). *Proc. Am. Assoc. Cancer Res.*, **33,** 7.

RAYNAUD FI, MCKEAGE MJ, WARD J, BERRY C, ODELL D, MURRER BA, SANTABARBARA P, JUDSON IR AND HARRAP KR. (1995). Pharmacokinetic study of orally administered ammine diacetato dichloro (cyclohexylamine) platinum (IV) (JM216) in a 5-day dose schedule Phase I clinical trial. In *Novel Approaches in Anticancer Drug design. Molecular Modelling and New Treatment Strategies.* Zeller WJ, D'Indalci M and Newell DR (eds). pp. 100–108. Karger: Basle.

RAYNAUD FI, MISTRY P, DONAGHUE A, POON GK, KELLAND LR, BARNARD CFJ, MURRER BA AND HARRAP KR. (1996). Biotransformation of the platinum drug JM216 following oral administration to cancer patients. *Cancer Chemother. Pharmacol.* (in press).

RICHON VM, SCHULTE N AND EASTMAN A. (1987). Multiple mechanisms of resistance to cis-diamminedichloroplatinum(II) in murine leukemia L1210 cells. *Cancer Res.*, **47,** 2056–2061.

SHARP SY, ROGERS P AND KELLAND LR. (1995). Transport of cisplatin and bis-acetato-ammine dichlorocyclohexylamine platinum(IV) (JM216) in human ovarian carcinoma cell lines: identification of a plasma membrane protein associated with cisplatin resistance. *Clin. Cancer Res.*, **1,** 981–989.

TWENTYMAN PR, WRIGHT KA, MISTRY PA, KELLAND LR AND MURRER BA. (1992). Sensitivity to novel platinum compounds in panels of human lung cancer cell lines with acquired and inherent resistance to cisplatin. *Cancer Res.*, **52,** 5674–5680.

VERSANTVOORT CHM, BROXTERMAN HJ, BAGRIJ T, SCHEPER RJ AND TWENTYMAN PR. (1995). Regulation by glutathione of drug transport in multidrug-resistant human lung tumour cell lines overexpressing multidrug resistance-associated protein. *Br. J. Cancer*, **72,** 82–89.